# Delivering PrePex Medical Male Circumcision Services Through a Mobile Clinic: The Experience From a Pilot Project in North West Province, South Africa

**DOI:** 10.1097/QAI.0000000000000798

**Published:** 2016-05-24

**Authors:** Tendesayi Kufa, Candice Chetty-Makkan, Mpho Maraisane, Salome Charalambous, Violet Chihota, Carlos Toledo

**Affiliations:** * The Department of Epidemiological Research, The Aurum Institute, Johannesburg, South Africa;; † The School of Public Health, University of the Witwatersrand, Johannesburg, South Africa; and; ‡ The Division of Global HIV/AIDS, The Centres for Disease Prevention and Control, Atlanta, GA.

**Keywords:** male, medical, circumcision, mobile, devices

## Abstract

We describe the implementation of a pilot project to demonstrate the safety and feasibility of providing PrePex circumcision from a mobile clinic. We analyzed available project diary entries and staff meeting minutes to identify challenges encountered. The main challenges identified were (1) daily time constraints because of setting up procedures, (2) transportation logistics for clients when the mobile clinic had moved to a different location, (3) integration and coordination of staff responsibilities, and (4) recruitment for PrePex services in the mobile clinic. The provision of PrePex device circumcision through a mobile clinic was feasible but careful planning and review of operational procedures were needed to resolve the implementation challenges.

## INTRODUCTION

In line with the World Health Organisation recommendations and guidelines for voluntary medical male circumcision (VMMC), South Africa has been providing VMMC services since 2010.^1^ To achieve global targets for HIV prevention, the country needs to circumcise 4.3 million males aged 15–49 years by 2016.^1^ By the end of 2013, 1.3 million men had been circumcised representing 31% of this target.^2^ Barriers to the rapid and effective scale up of VMMC services in the country included shortage of adequately trained staff and equipped facilities required to perform circumcisions, especially in hard-to-reach areas.^3^ In South Africa, only doctors are allowed to perform the minor surgical procedure required for circumcision, and there is no policy allowing task-shifting to nurses. Another barrier has been the suboptimal demand of VMMC service by men, with fewer of them accessing circumcision services because of fear of pain, reluctance to abstain from sex, and concerns about missing work during the recovery period.^4–6^ The use of mobile VMMC clinics could enhance access to services in hard-to-reach areas and address challenges related to limited clinical staff, as nurses may perform the PrePex procedure. Additionally, the PrePex circumcision method takes into account concerns about pain, injections, and missing work.^7^ There are limited data on the feasibility of providing VMMC services through mobile clinics and the challenges faced by providers in providing these services. We describe our experience of providing VMMC services using the PrePex device in the context of a pilot project implemented in Ngaka Modiri Molema district of the North West province in South Africa. Here, we present findings related to the feasibility of providing PrePex through a mobile clinic and the challenges encountered (primary safety outcomes of the evaluation to be presented elsewhere). Documenting these experiences, particularly the challenges, is helpful in informing the scale up of mobile MMC services.

## METHODS

### Description of the Pilot Project and Mobile Clinic

**FIGURE 1.**
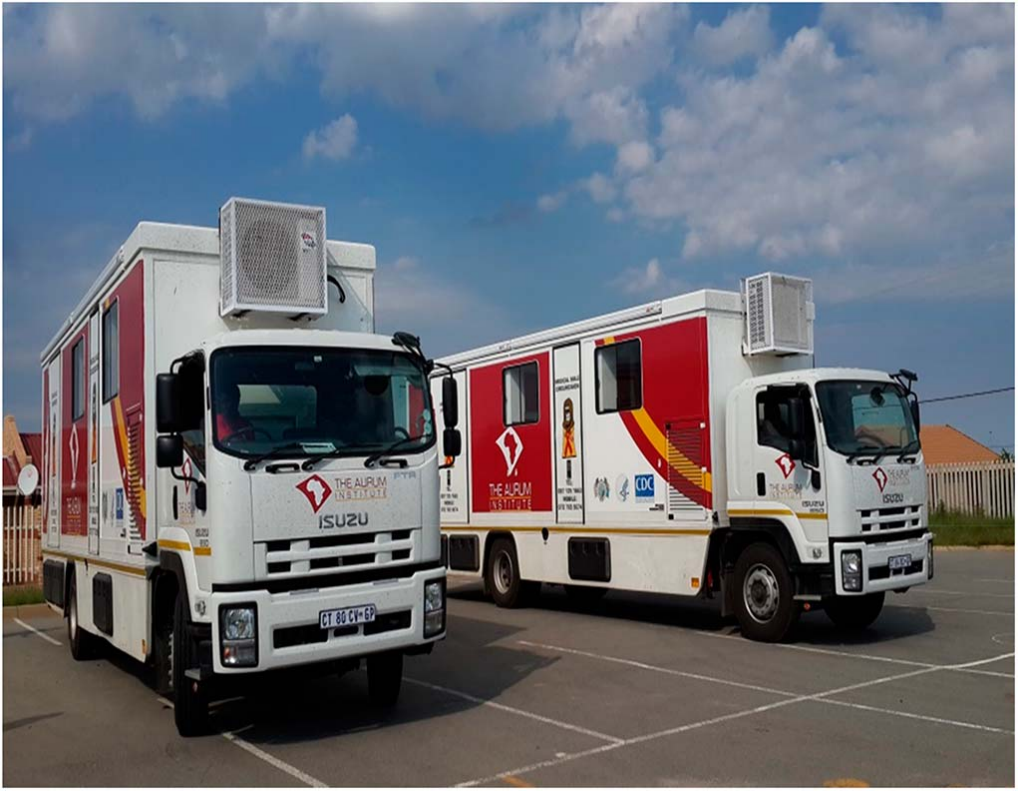
Image of the mobile vans.

This PrePex pilot project was conducted in urban and periurban areas of Ngaka Modiri Molema district. Before implementation of this PrePex pilot project, surgical VMMC services had been provided by a team of providers rotating and operating at fixed Department of Health facilities in the district. The aim of the project was to evaluate the safety and feasibility of providing VMMC services using the PrePex device from a mobile clinic. The mobile clinic comprised 2 custom built vans (Fig. 1) designed to allow assessment of clients, provision of surgical and PrePex device circumcisions, and the follow-up of clients. The mobile clinic was deployed to move between 5 fixed department of health facilities, where there were no VMMC services available and that were within a 50-km radius from the central office, where it was parked at night. The mobile clinic would return to the same location to conduct surgical and PrePex device circumcisions for 1–3 days at a time depending on demand.

Before the mobile clinic moved to the next health facility, recruiters were deployed ahead of time to conduct community-based VMMC and PrePex circumcision awareness activities (which included presentations at health care facilities, companies, taverns, sporting events, community events, and door-to-door activities), recruit potential clients, and make bookings for circumcisions. When clients arrived at the mobile clinic, they were assessed for VMMC eligibility and given the option to be circumcised using the PrePex device or surgical circumcision. PrePex device circumcisions were conducted according to the set evaluation protocol, whereas surgical circumcisions were provided according to routine programmatic guidelines. To be eligible for Prepex device circumcision, male clients had to be aged between 18 and 49 years, HIV uninfected, previously uncircumcised, and have no contraindications for circumcision using the device. After device placement, clients were contacted telephonically on day 2 after placement for an interview and were reminded to return to the mobile clinic for review on day 4. Clients were also asked to return to the mobile clinic for device removal on day 7 after placement. If the mobile clinic had moved to another location, clients were transported from the location of the mobile clinic at their placement to the new location. No transport was provided for clients if removals were scheduled at the same location where placements took place. After device removal, clients were followed-up through 2 clinic visits (at days 14 and 42 after placement, respectively) and 3 telephone contacts (at days 21, 28, and 35 after placement) until complete epithelial healing or until day 70 after device placement, whichever came first. Transport was also provided for the days 14 and 42 after placement visits, when necessary. At the placement and removal visits, clients were provided with counseling on how to care for themselves with the device in situ and after removal. After placement or removal, care instructions were reinforced at every visit or contact with the client.

The pilot project was divided into 2 phases. The first phase was focused on training and was implemented under the supervision of a provider credentialed by the device manufacturer as a master trainer. This phase was intended to last 2 weeks or to continue until 4 doctors and 2 nurses were certified as competent PrePex providers. The second phase of the pilot project was the evaluation of acceptability, safety, time to healing, and experiential feasibility and started a month after the training phase ended. The aim of this phase—intended to last 8 weeks—was to circumcise and follow-up 550 clients using PrePex to evaluate outcomes. During the second phase, PrePex device placements and removals were conducted by the 4 doctors and 2 nurses trained during the training phase. Two additional nurses, trained through another PrePex project, joined the team half way through the second phase. Each device placement or removal required 2 PrePex certified providers, where one provider conducted the placement or removal while the other provider assisted.

### Data Collection and Analysis

The project coordinator, a nurse and Prepex trained provider or her designee, was responsible for ensuring that project diary entries and logs were completed and that project meeting minutes were documented. For this analysis, we reviewed available project diary entries, screening and enrollment logs, and meeting minutes to identify documented challenges that occurred during PrePex VMMC implementation from a mobile clinic. A qualitative inductive approach was used to identify common key themes that emerged from the records and themes, which were then coded and summarized.

## FINDINGS

In total, 890 potential clients were assessed for eligibility and 641 (72%) clients had circumcision using the PrePex device. During the training phase, 150 clients were assessed and 90 (60%) clients were circumcised using PrePex, whereas 740 clients were assessed and 551 (74%) clients circumcised in the evaluation phase. Of the 60 assessed in the training phase but not circumcised with PrePex, 29 (48.3%) refused PrePex circumcision, whereas the remainder were not eligible for device placement because of contraindications to device placement; all 60 were referred for surgical circumcision. Of the 189 screened in the evaluation phase but not circumcised with PrePex, 79 (41.8%) refused PrePex circumcision, whereas the remainder had contraindications for device placement; all 189 were referred for surgical circumcision. All 641 (100%) clients who had the Prepex device placed returned to the mobile clinic for device removal. The follow-up rates for days 4, 14, and 42 (or healing status assessment visit) were 65.3%, 53.9%, and 72.4%, respectively. There were 18-weekly staff diary entries available for analysis out of 27 weeks of pilot project implementation and minutes from 10 team meetings were available for review. Some diary entries were missing because they were not entered and minutes were not documented. Most project diary entries were from the period April to July 2014, the first half of the evaluation phase.

Table 1 presents the main challenges documented by the team and identified in the analysis and the steps taken to resolve them. These challenges included the following: (1) daily time constraints due to set-up procedures; (2) transportation logistics for clients when the mobile clinic had moved to a different location at the time removal was required; (3) integration and coordination of staff responsibilities by circumcision method; and (4) recruitment for PrePex VMMC services in the mobile clinic.

**TABLE 1.**
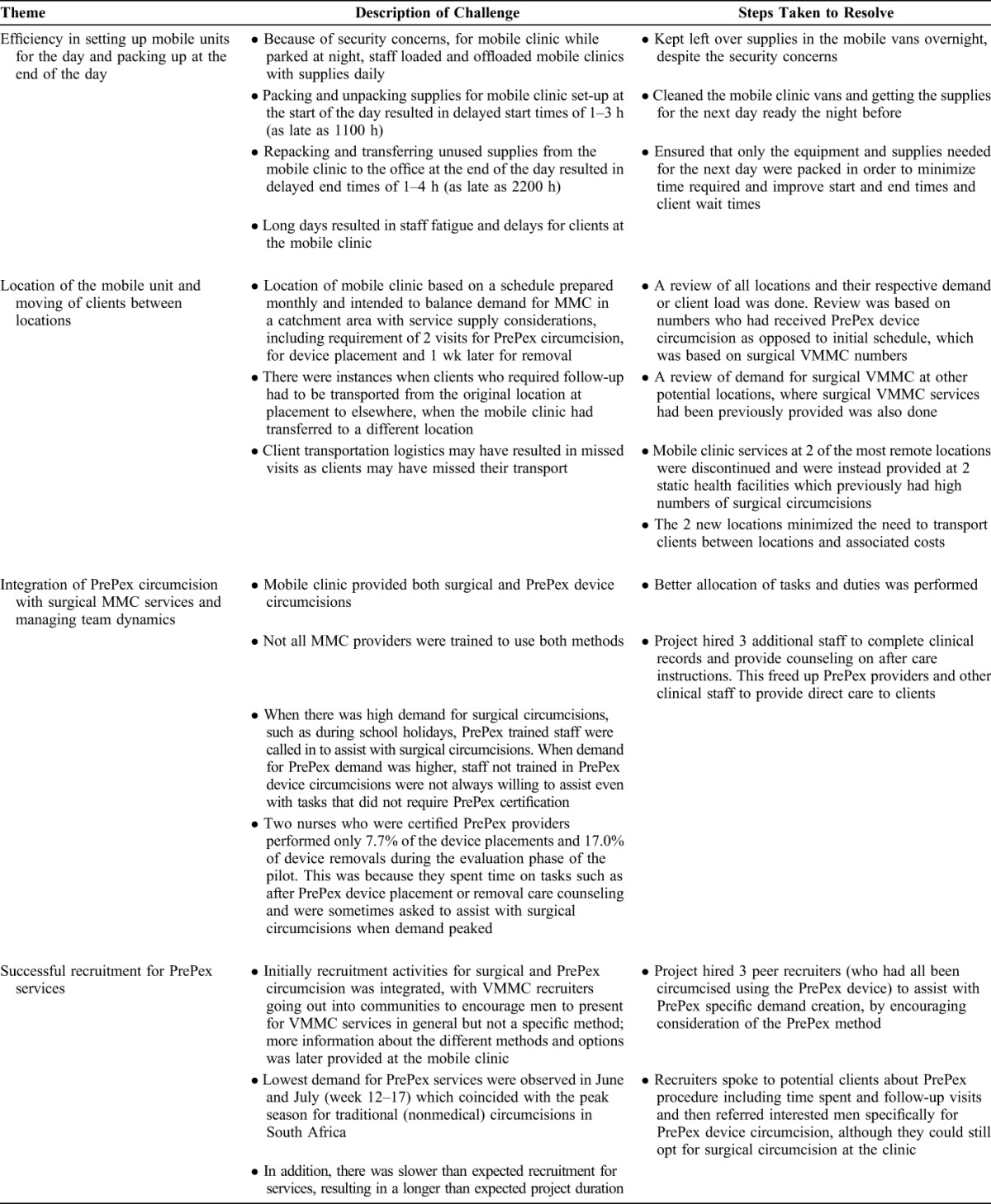
Challenges Documented and Steps Taken to Resolve Them

Daily time constraints due to setting up procedures and the delays in daily set-up of the mobile clinic resulted in limited time to provide services and sometimes longer wait times for clients. To overcome this challenge, the team left supplies in the mobile clinics overnight despite the security concerns. Another solution that could have been adopted to overcome this challenge would be streamlining the inventory list for mobile clinics to minimize quantities which require packing.

Transporting clients between application and removal locations if the mobile clinic was no longer locally available at the time of removal was costly and complicated the logistical arrangements for the project. The need to transport clients between locations added to the total length of time clients spent to access the service. Although the team was not able to fully resolve this challenge, having separate teams doing removals and placement at different locations at the same time and staying in 1 location long enough to complete all removals were possible solutions that were attempted.

With respect to the integration and coordination of staff responsibilities by circumcision method, pilot project team comprised doctors, nurses, and other support staff who were not all trained to provide PrePex device circumcisions. This created challenges with allocating tasks when there was high demand for both device and surgical circumcisions. The team overcame this challenge by improving allocation of tasks and getting additional staff to assist with counseling and other administrative duties. Training of all staff in both surgical circumcision and PrePex device circumcision techniques is a possible solution the team could have used to ensure efficient use of available staff.

Regarding recruitment for PrePex circumcision, the team was unable to meet the 10-week goal for completing the number circumcisions required for the pilot project. In addition, the recruitment for PrePex fluctuated from week to week and was lowest during the period when the demand for circumcisions was expected to be highest. The use of peer recruiters (men who had been circumcised using the PrePex device) and implementing PrePex-specific recruitment activities were 2 strategies used by the team to improve demand. Because these interventions were implemented late in the pilot, it was not clear what impact they had on the number of device-based VMMCs done, although their use could be further evaluated in other programs.

## DISCUSSION

This article describes the experiences and operational challenges documented during implementation of a PrePex pilot project. The pilot project showed that although Prepex circumcision can be successfully provided through mobile clinics, careful planning and continuous review of operational plans and challenges are required. The experiences described in this article are relevant to projects providing both surgical and circumcision with devices such as Prepex from mobile clinic settings and may not readily apply to other settings. Delays in setting up for the day and slow recruitment are challenges that could be experienced in mobile clinics providing VMMC services regardless of method used. However, providing PrePex device circumcision through a mobile clinic may be more challenging than providing surgical circumcisions in similar settings because of the need to coordinate device placements, device removals, and follow-up. As the pilot project required more frequent after circumcision visits than would be required in routine programmatic settings, some operational challenges encountered may not apply to routine programmatic settings. Finally, the incomplete documentation of project diary entries and project team minutes may have resulted in some challenges being undocumented and therefore missed.

In conclusion, providing VMMC using the PrePex device in a mobile clinic was feasible although there were a number of operational challenges that needed to be addressed. Careful planning and continuous review of operational plans and the challenges encountered are needed to manage logistical aspects of such projects and creating demand.
